# Culture-dependent and independent approaches for identifying novel halogenases encoded by *Crambe crambe* (marine sponge) microbiota

**DOI:** 10.1038/srep02780

**Published:** 2013-09-27

**Authors:** Başak Öztürk, Lenny de Jaeger, Hauke Smidt, Detmer Sipkema

**Affiliations:** 1 Wageningen University, Laboratory of Microbiology, Dreijenplein 10, 6703 HB Wageningen, The Netherlands

## Abstract

Sponges harbour microbial communities that contribute to the genetic and metabolic potential of their host. Among metabolites produced by sponge-associated microbial communities, halogenated compounds are of special interest because of their biotechnological potential. In this study, we have examined the diversity of the cultivable fraction of the marine demosponge *Crambe crambe* microbiota. Application of complementary cultivation methods yielded 107 bacterial isolates, some of which may be sponge-specific based on their phylogenetic analysis. Among these, *Psychrobacter* sp. was found to contain a putative halogenase gene. In addition to the culture-dependent approach for discovering halogenase genes, a cDNA library was constructed to determine the diversity of halogenase genes expressed *in situ* by the *C. crambe* microbiota. To this end, seventeen putative tryptophan halogenase cDNA sequences were identified, most of which were only remotely related to known halogenase genes, indicating the potential for novel bioactive compounds being produced by the *C. crambe* microbiota.

Sponges are sessile, filter-feeding aquatic animals of the phylum Porifera whose fossil records date back to the Precambrian period^1^. These unique animals harbour microbial communities, which vary in density and diversity in different host species^2^,^3^. The microbial communities associated with marine sponges can contribute significantly to the local and global element cycles and primary production^4^,^5^ as well as potentially play an additional role in the host's carbon, nitrogen and sulphur metabolism^6^. These microbial communities have also been hypothesized to contribute to the host metabolism through the production of secondary metabolites^7^,^8^,^9^. These secondary metabolites have attracted attention due to their potential applications in biotechnology and pharmaceutical industry.

FADH_2_-dependent halogenases are the largest class of halogenases identified to date, and encoding genes have been found in every gene cluster for production of halogenated metabolite production so far, including those producing compounds with antibiotic properties^10^. FADH_2_-dependent halogenases can be distinguished into three classes: tryptophan halogenases, phenol halogenases and pyrrole halogenases depending on their preferred substrate^11^.

A recent study by Bayer et al.^12^ focused on the culture-independent discovery and characterization of FADH_2_–dependent halogenase genes. They found several sponge-specific clusters of FADH_2_–dependent halogenase gene sequences that were phylogenetically distinct from those previously known. While such cultivation-independent studies are indispensable for the discovery of genes in bacteria which cannot be cultivated, it is beneficial to complement these approaches with cultivation-based strategies for a number of reasons, including the ability to be able to validate the function of these genes in their original host, assess their role in symbiont physiology and host-microbe interactions, as well as to determine potential biotechnological applications.

Cultivation of sponge symbionts is notoriously difficult, and the previous attempts to cultivate sponge-associated bacteria have recovered not more than 14% of the total sponge-associated microbial community^13^,^14^,^15^,^16^. To increase the chances of meeting the metabolic requirements of previously uncultured bacteria, media with different compositions and nutrient concentrations should be used^13^. Such cultivation-based approaches have previously been used to isolate sponge-associated bacteria with antimicrobial activities^17^,^18^,^19^. To our knowledge, there have been no attempts to cultivate sponge-associated microorganisms which produce halogenated compounds.

To close this knowledge gap, the aim of the current study was to discover novel halogenase genes and identify the members of the sponge microbial community that potentially produce halogenated molecules. To this end, we focused on the diversity and expression of FADH_2_-dependent halogenase genes within the microbial community associated with the marine demosponge *Crambe crambe*. This sponge contains microbial communities with a low density and diversity^20^,^21^ and can therefore be considered an ideal model to link metabolic functions of interest to specific microorganisms. *C. crambe* has been previously shown to produce biotechnologically interesting metabolites called crambescidins^22^,^23^ but the potential of the microbial community to produce these and other compounds has not been assessed. We have approached this question using a complementary set of culture-dependent and independent methods. Several cultivation strategies were employed to isolate *C. crambe* associated microorganisms. The isolates were screened by PCR with degenerate primers targeting conserved regions of FADH_2_-dependent halogenase encoding genes^24^. To complement cultivation-dependent studies, bacterial diversity in *C. crambe* was analysed by pyrosequencing of 16S rRNA gene amplicons. In addition a cDNA library was constructed to determine the in situ expressed fraction of FADH_2_-dependent halogenase genes.

## Results

### Cultivable assemblage and phylogenetic analysis of *C. crambe* associated microorganisms

**Figure 1:**
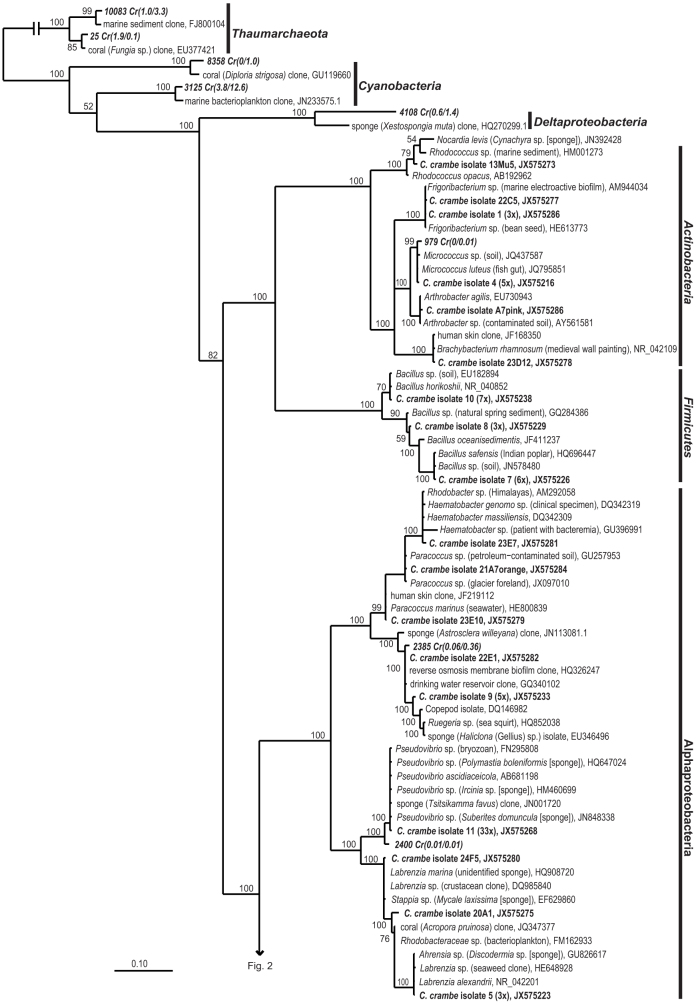
Bayesian phylogram based on 16S rRNA gene sequences from *C. crambe* isolates (in bold), selected pyrosequencing-based OTUs of *C. crambe* associated microorganisms (in italic) and nearest neighbours. When isolate-derived OTUs consist of more than one isolate, a representative sequence is included in the tree and the number of isolates represented by the OTU is included in parentheses. For cultivation independent OTUs the relative abundance in Crambe 1 and Crambe 3, respectively, is included in parentheses. The numbers above or below the branches correspond to posterior probability (PP) values of the Bayesian analysis. The scale bar corresponds to the mean number of nucleotide substitutions per site. Beta- and Gammaproteobacterial isolates and cultivation-independent OTUs are shown in Fig. 2.

**Figure 2:**
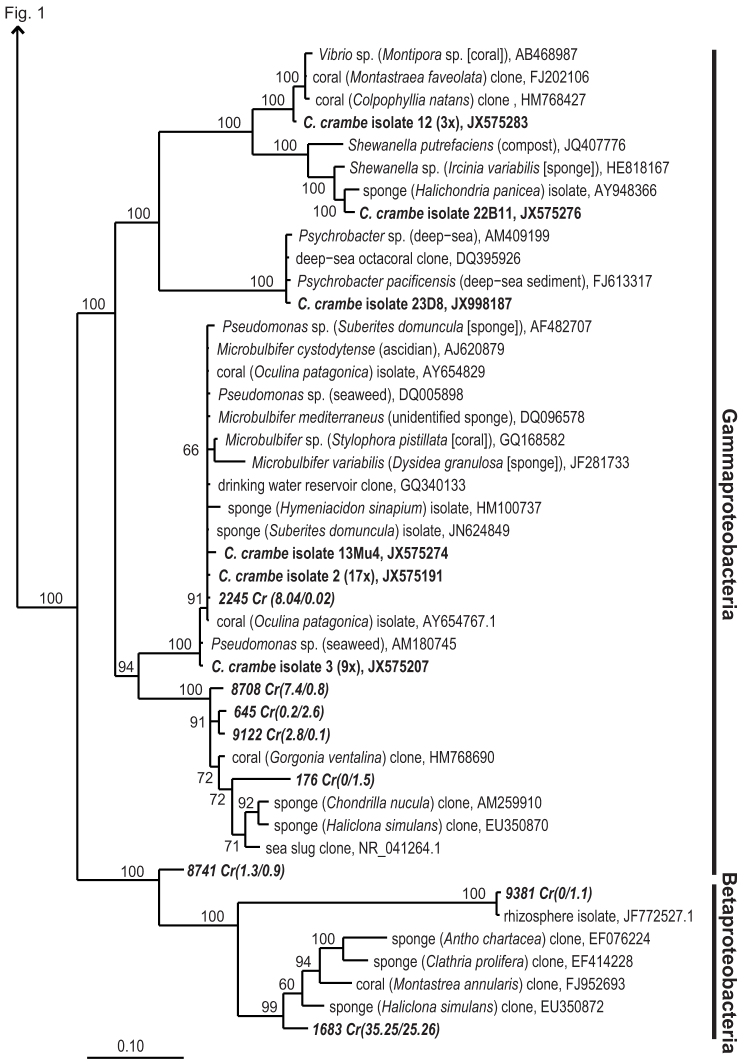
Bayesian phylogram based on 16S rRNA gene sequences from Beta- and Gammaproteobacterial *C. crambe* isolates. For a description of the Figure organisation, see Fig. 1.

A total of 107 bacterial isolates were obtained from three *C. crambe* individuals, referred to as Crambe 1, Crambe 13 and Crambe 19, and using a broad range of culture media that differed in nutrient composition and concentration. The most frequently isolated genus was *Pseudovibrio* (33 isolates), followed by *Microbulbifer* (27 isolates), *Bacillus* (16 isolates) and *Ruegeria* (6 isolates). Other genera isolated in smaller numbers included *Paracoccus* (3 isolates), *Vibrio* (3 isolates), *Shewanella* (1 isolate), *Psychrobacter* (1 isolate), *Arthrobacter* (1 isolate), *Micrococcus* (1 isolate), *Frigoribacterium* (1 isolate) and *Rhodobacter* (1 isolate). *Pseudovibrio* spp. were isolated from media 1–5, while *Ruegeria* spp. were cultivated only on media 1 and 3 (See Methods: Media used for cultivation experiments). *Bacillus* spp. were cultivated on rich media (1 and 5) and *Microbulbifer* spp. could be retrieved from media 10, 11, 12 and 14. *Psychrobacter* sp. which was later found to have a halogenase gene (isolate 23D8), was cultivated on the rich medium M13 0.5 × (medium 2). All isolates grew well on solid and liquid media. The low number of isolates obtained compared to the high number of media and conditions tested did not allow a statistically sound assessment of the impact of cultivation conditions on the types of isolates obtained. Phylogenetic analysis based on 16S rRNA gene sequence (Figure 1 and 2) showed that *Pseudovibrio* sp. isolated from *C. crambe* (Crambe isolate 11) is closely related to microorganisms previously isolated from a wide range of marine sponges^17^,^18^,^19^,^25^. *Microbulbifer* spp. (Crambe isolates 2 and 3, and isolate 13Mu4), *Ruegeria* spp. (Crambe isolates 9 and 22E1) and *Shewanella* sp. (Crambe isolate 22B11) are also closely related to microorganisms previously detected in marine sponges and other marine invertebrates by cultivation dependent and independent methods^13^,^26^. In addition, *Vibrio* sp. (Crambe isolate 12) is closely related to bacteria previously isolated from corals^14^,^27^. Actinobacteria and Firmicutes cultivated in this study have no near neighbours that were previously isolated from marine sponges or other marine invertebrates and are unlikely to be truly sponge symbionts, although this cannot be excluded at this time since corresponding sequences are also not commonly found in seawater.

### Relative abundances of the cultivated microorganisms in the *C. crambe* microbiota

The *C. crambe* microbiota as identified by cultivation-independent methods was dominated by a sponge- and coral-specific betaproteobacterium (Figure 2). Based on the pyrosequencing data, this bacterium accounted for 31.4 ± 7.3% of the reads of which the majority was assigned to one OTU (OTU1683). However, either due to intraspecific variation, presence of multiple non-identical copies, or sequencing errors, 18 other closely related OTUs were found (data not shown). Although OTU1683 makes part of a clade of sponge- and coral-derived betaproteobacteria (for further details on the positioning of this OTU within the cluster SC112, see cr050 and cr065 in Simister et al., 2012^28^), it shares only 94% identity with its nearest neighbour, a clone sequence derived from the marine sponge *Antho chartacea* (EF076224.1).

To determine the relative abundances of the isolates in the *C. crambe* microbial community avoiding cultivation bias, each 16S rRNA gene sequence obtained from the isolates was compared to pyrosequencing libraries generated. A total of 14755 non-chimeric reads were evaluated, and the number of occurrences for each local blast hit was counted. Although some isolates were detected by culture-independent methods, few were present at a relative abundance of more than 1% of the reads (Table 1). *Microbulbifer* sp. (isolates 2, 13Mu4) was present in relatively high numbers in Crambe 1 from which it was isolated, but not in the other individual Crambe 3. The dominant betaproteobacterial symbiont did not yield to cultivation.

### Diversity of halogenase genes

Total RNA was extracted from *C. crambe* tissue to produce cDNA libraries using RT-PCR amplification with tryptophan halogenase-specific degenerate primers. Seventeen putative halogenase gene sequences were identified from this library. In contrast, using the same primers, only one of the 107 isolates (isolate 23D8, *Psychrobacter* sp) was found to possess a putative halogenase gene. By comparison to the NCBI database of microbial proteins and the Conserved Domains Database^29^, all of these the retrieved sequences were classified as putative tryptophan halogenases.

**Figure 3:**
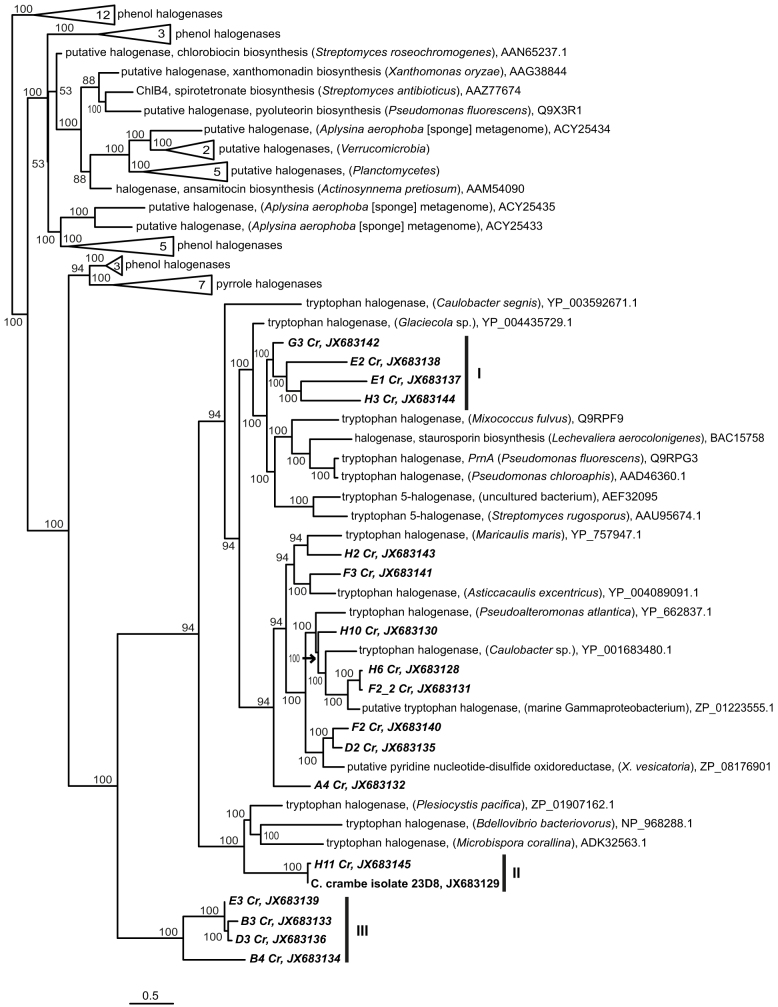
Phylogenetic analysis of predicted halogenase sequences. The phylogenetic tree was constructed from 81 amino acid positions using RaxML Maximum Likelihood tree calculator. Sequences derived from this study are shown in bold. The tree has been drawn to scale and Bootstrap values greater than 50 are shown (100 replicates). Tree was rooted with the haloacetate dehalogenase from *Streptomyces globisporus* (AAL06675, not shown). The scale bar corresponds to the mean number of amino acid substitutions per site on the respective branch. NCBI accession numbers of reference sequences as well as those obtained in this study are indicated.

Phylogenetic analysis of the *C. crambe* microbiome-associated halogenases was performed based on amino acid sequence (Figure 3). Three clades of *C. crambe*-derived putative halogenases could be distinguished of which especially clade III was only remotely related to other previously-reported sequences. Only four sequences were not classified into any of these clades. The predicted halogenase sequence from the *C. Crambe* isolate 23D8 has 100% similarity to a cDNA sequence (H11 Cr) on amino acid level and is likely to be the same gene. Overall the halogenase cDNA sequences clustered together with the other tryptophan halogenase sequences in the tree, confirming their annotation. The full halogenase sequences obtained from *Aplysina aerophoba* by Bayer et al.^12^ were phylogenetically distant from the sequences obtained from *C. crambe* microbiota.

## Discussion

Phylogenetic analysis of the *C. crambe* microbiome using complementary cultivation-dependent and cultivation-independent molecular approaches indicated a low microbial diversity, which is dominated by one yet uncharacterized bacterium that belongs to a betaproteobacterial clade that is specific to sponges and corals. This is in accordance with previous knowledge that *C. crambe* is a low bacterial diversity and density sponge^20^,^21^. Data presented here suggest that, cultivation approaches introduce a certain bias. Some of the isolates were also detected in the 16S rRNA gene pyrosequencing libraries, however, only in low numbers, amounting to 0.4% of the total microbiota, while others were absent. One exception were isolates most closely related to *Microbulbifer* spp., which were present in high numbers only in one individual and absent in the other, based on the pyrosequencing data. Although this may point towards *Microbulbifer* spp. not being part of the core *C. crambe* microbiota, the fact that they were found in relatively high numbers in the individual from which it was isolated shows an overlap between cultivation dependent and independent approaches to a certain extent. *Pseudovibrio* (*C. crambe* isolate 11), which was the most frequently isolated genus, was detected in only very low numbers in both 16S rRNA gene sequence datasets. Close relatives of this bacterium have been frequently isolated from a wide range of sponge species, and their presence in the sponge tissue was demonstrated by Fluorescence In Situ Hybridization^26^,^27^ though they were rarely detected in 16S rRNA clone libraries. Phylogenetic analysis of our isolate confirms this observation, as almost all the 16S rRNA gene sequences closely related to those of our isolates originate from other sponge isolates, rather than being directly amplified from sponge DNA. *Pseudovibrio* spp. isolated from other marine sponges have been described to produce bioactive compounds, especially substances with antibiotic properties^17^,^18^,^19^,^25^. Taking their low numbers into account, however, we cannot draw any conclusions on the role of these organisms in the host's chemical defence against harmful bacteria or predators. On the other hand, the potential production of antibacterial compounds by these bacteria may be one of the reasons why they overgrow enrichment cultures. This should be taken into account in future cultivation studies. Among the other isolates, those clustering within the genera of *Ruegeria*, *Microbulbifer* and *Shewanella* are closely related to microorganisms that were previously detected in other marine sponges and therefore could be consistent sponge-associated bacteria.

Taking the low microbial diversity and density of *C. crambe* into account, the number of cDNA sequences that were annotated as halogenase genes in this study is surprisingly high. This finding is especially interesting as FADH_2_-dependent halogenase genes were not found in the previously-investigated low-microbial-abundance sponge species^12^. We have observed six halogenase sequences/clades in addition to the three distinct *C. crambe* halogenase clades. Unlike the previous study by Bayer et al.^12^, our cDNA sequences did not cluster into one sponge-specific clade. The homologies of the sequences reported in this study to their nearest relative varied from 80% down to 40% based on amino acid identity, suggesting that these genes are quite different from any of the previously described halogenases and could take part in producing yet uncharacterized halogenated compounds. These low similarities to amino acid sequences in public databases also means that reliable taxonomic classification of the halogenase cDNA sequences could not be made except for cDNA sequence H11 Cr, which had 100% similarity to the halogenase gene sequence amplified from isolate 23D8. When the *C. crambe* metagenome sequence becomes available, taxonomic assignment of these genes may become more achievable.

The 16S rRNA gene sequence of isolate 23D8, which is the only isolate from which a halogenase gene was detected by PCR, was most closely related to those of *Psychrobacter* spp. Members of this genus have previously been described to harbour bioactive compounds and dehalogenases^29^,^31^. The halogenase gene from this isolate has a very high sequence homology to one of the cDNA sequences obtained from the microbial community of *C. crambe*, indicating that the gene could be detected by culture dependent and independent methods and is expressed within the microbial community, although *Psychrobacter* sp. does not make up a large fraction of the microbial community.

All cDNA sequences as well as the halogenase sequence obtained from the isolate 23D8 were annotated as tryptophan halogenases. It has previously been reported that tryptophan halogenase genes are involved in the production of a variety of structurally very different compounds when co-expressed with respective biosynthetic gene clusters^30^. Therefore, it is possible that these genes were detected in the cDNA libraries because they are expressed in high levels by the microorganism to contribute to the production of a set of different metabolites.

The ecological and metabolic significance of the expressed halogenase genes remains unclear. In single bacterial species, the role of these secondary metabolites is unknown and they are normally produced at levels that are hardly detectable^32^. Therefore, it is hard to imagine that these halogenated compounds would have a benefit to the sponge host in terms of inhibiting the growth of potentially harmful microorganisms even if they have antibiotic properties at higher concentrations. However, the functional characterization of these genes combined with future genomic and transcriptomic studies can provide us with further insights into the role of halogenated compounds in the sponge holobiont, and can contribute to the discovery of new compounds of biotechnological and pharmaceutical interest.

## Methods

### Sample collection and processing

Individuals of the Mediterranean sponge *C. crambe* were collected by SCUBA diving: Crambe 1 and Crambe 3 at Punta Santa Anna in Blanes, Spain (N41°40′23.46″ E2°48′10.80″) at a depth between 10 and 15 m on June 5th, 2008, and Crambe 13 and Crambe 19 offshore L'Escala, Spain (N 41° 40′ 23.46″ E 2° 48′ 10.80″) on January 15th, 2012, at a depth of approximately 15 m. Specimens were brought to the surface in ziplock plastic bags. The sponges were rinsed two times with sterile artificial seawater (natural sea salt mix, Oceanic Systems, Dallas, TX, USA) before grinding the tissue with a sterilized mortar and pestle. Two tissue volumes of sterilized artificial seawater (ASW) were added to obtain a homogeneous cell suspension. For specimen Crambe 1 the cell suspension was divided in aliquots of 1.2 ml and mixed with 0.6 ml 50% sterile glycerol in ASW. The samples were frozen until –20°C before they were stored at –80°C. Separate pieces of tissue from Crambe 1 and Crambe 3 were placed in 96% ethanol after washing with ASW. The ethanol was completely replaced after 1 and 2 days. Samples Crambe 13 and Crambe 19 were used immediately upon collection to initiate cultivation experiments. Natural Seawater (NSW) was collected near Zierikzee, The Netherlands.

Tissue samples that were used for RNA extraction were sliced with a sterile scalpel and immersed in 10 volumes of RNALater (Ambion, Bleiswijk, the Netherlands). Samples were kept at 4°C for 24 hours and subsequently frozen at −20°C. The time between sample acquisition and fixation was no longer than 20 minutes.

### Media used for cultivation experiments

Sixteen different media were used for isolation of bacteria: 1) M13 1 × (modified after^47^): Artificial Seawater (ASW)^48^, peptone (0.025% w/v), yeast extract (0.025% w/v), casamino acids (0.075% w/v), glucose (0.02% w/v), Hutner's Basal Salts (HBS) (2% v/v), 0.1 M Tris-Cl (5% v/v), vitamin solution no 6 (0.1% v/v), 2) M13 0.5 ×: M13 1 × with half-strength ASW, 3) OM 1 × (modified after^49^): ASW, HBS (2% v/v), 0.1 M Tris-Cl (5% v/v), vitamin solution no 6 (0.1% v/v), 200 μM of each 20 amino acid, Tween 20 (0.002% w/v), glucose, pyruvate, citrate, succinate and 2-oxoglutarate (200 μM each, Na^+^-salts) and a fatty acid mixture with formate, acetate and propionate (200 μM each, all Na^+^-salts), 4) OM 0.5 ×: OM 1 × with half-strength ASW, 5) DMEM medium (modified after^50^): ASW, DMEM (Gibco 52100-021, powder, high glucose) (0.05% w/v), phytohemagglutinin (Gibco®) (0.15% v/v), HBS (2% v/v), 0.1 M Tris-Cl (5% v/v), 6) Diverse poor agar: 0.011% w/v of each D-maltose, mannitol, D-glucose, potato starch, galactose, peptone, tryptone and yeast extract in NSW, 7) 1% Marine broth agar: Marine broth (BD Difco™ 2216) (0.037% w/v), phenol red (0.0017% w/v) in NSW, 8) Peptone Starch agar^13^, 9) Bordet-Gengou blood agar (modified after^51^): Laked horse blood (1.5% v/v, Oxoid), glycerol (0.2% v/v), peptone (0.2% w/v), potato starch (0.09% w/v), NaCl (0.25 w/v) in NSW, 10) Fluid Thioglycollate agar^13^, 11) Alcaligenes isolation agar^52^: peptone (0.01% w/v), meat extract (0.06% w/v), NaFe(III)-EDTA (0.03% w/v), lactate-succinate solution (5% v/v) (1.3% w/v succinate, 0.46% v/v lactic acid in NSW) in NSW, 12) Mucin agar: Mucin (Sigma) (0.01% w/v) in NSW, 13) Tryptone soya agar: Tryptone soya agar (Oxoid) (1.5% w/v) in NSW, 14) Rhodocyclus isolation agar (modified after^52^): Yeast extract (0.01% w/v), NH_3_ solution (1% v/v) (5% NH_3_-acetate in NSW), NH_4_ solution (1% v/v) (5% (NH_4_)_2_SO_4_ in NSW), Ferric citrate solution (5% v/v) (0.2% w/v Iron(III) Citrate), vitamin B_12_ solution (0.1% v/v) (2 mg/L) in NSW, 15) Actinomycete isolation medium^13^, 16) Colistin marine agar (modified from^53^) NSW (90% v/v), demineralized water (9.625% v/v), Marine broth (0.375% w/v), colistin (7.5 mg/L). Solid media were obtained after supplementing the liquid media with 1.5% Bacto-Agar. HBS was prepared according to Cohen-Bazire^54^ and the vitamin solution no. 6 was prepared according to Staley et al.^55^. The pH values of all media were adjusted to 7.0. To all media 6–16, the following supplements were added: 0.01% v/v trace metal and phosphate solutions^14^, and 0.01% v/v of Basal Medium Eagle Vitamin Solution 1000 ×.

### Cultivation experiments

Bacteria were isolated from *C. crambe* by using both liquid and solid cultivation media. For all cultures, 50 μl of the homogenate (Crambe 13 and 19) or cryopreserved cell suspension (Crambe 1) was used to inoculate 5 ml aliquots of each liquid media in order to make enrichment cultures, or agar plates. Media 1–5 were used for the cultivation experiments from the pooled fresh samples of Crambe 13 and 19, and media 6–16 were used for cultivation experiments from frozen samples of Crambe 1. Ten replicates were inoculated for each solid or liquid medium. Cultures were incubated at 20°C for one week before isolation of microorganisms by dilution-to-extinction or single-colony plating. Agar plate cultures were monitored for an additional 5 weeks to observe slow-growing or metabolically-biased species. To isolate microorganisms by the dilution-to-extinction method, 180 μl of each medium was dispensed into 96-well microtiter plates, and 20 μl of each liquid enrichment culture was inoculated into 8 wells of each microtiter plate. Dilution series were generated in eight parallels by 10-fold dilutions in 12 consecutive wells. The dilution series were repeated three times with the highest dilution that showed growth. For isolation of microorganisms on agar plates, each single colony was plated re-streaked onto a new agar plate and isolated by transferring single colonies on fresh agar plates three times. The purity of each culture was verified by microscopy.

### DNA extraction and amplification

DNA of the isolates was extracted with the CTAB-lysozyme method^33^. Amplification of the 16S rRNA genes was performed using the GoTaq® Hot Start Polymerase kit (Promega, Leiden, the Netherlands) with universal bacterial primers 27F and 1492 R^34^. The PCR conditions were: initial denaturation (5 min, 95°C) followed by 30 cycles of denaturation (30 s, 95°C), primer annealing (40 s, 52°C), primer extension (90 s, 72°C), and a final extension (5 min, 72°C). Reactions (50 μl) contained 1 × GoTaq® Green Flexi buffer, 1.5 mM MgCl_2_, 0.2 mM of each dNTP, 0.2 μM of each primer, 1.25 u GoTaq® Hot Start Polymerase and 100 ng template DNA. Purification of PCR products, determination of sequences using the 16S rRNA gene-specific primer 27F and quality check were performed by GATC Biotech, Konstanz, Germany.

DNA extraction of sponge tissue samples from Crambe 1 and Crambe 3 preserved in ethanol was done according to the tissue extraction protocol from the DNeasy blood & tissue kit (Qiagen, Hilden, Germany) five days after collection. Tag-encoded amplicon pyrosequencing was conducted with universal prokaryote primers PRK341F (CCT AYG GGR BGC ASC AG) and PRK806R (GGA CTA CNN GGG TAT CTA AT) to amplify an approximately 466 bp fragment of the 16S rRNA gene comprising the V3 and V4 regions (İnceoğlu et al., 2011). PCR amplification was performed in a volume of 40 μl using 1 × Phusion HF buffer, 2.5 mM MgCl_2_, 0.2 mM dNTP mixture, 0.8 U Phusion Hot Start DNA polymerase (Finnzymes, Espoo, Finland), 0.5 μM of each primer and 1 μl template DNA. PCR was performed using the following conditions: an initial denaturation at 98°C for 30 s, followed by 30 cycles of denaturation at 98°C for 5 s, annealing at 56°C for 20 s, elongation at 72°C for 20 s, and a final elongation at 72°C for 5 min. After PCR amplification, the samples were held at 60°C for 3 min and then placed on ice before they were checked on a 1.25% (w/v) agarose gel and purified using the Millipore DNA Gel Extraction Kit (Millipore, Billerica, MA, USA). A second round of PCR was performed as described above, except that a pyrosequencing adapter (CCT AYG GRB GCA SCA G) and 8 different barcodes of 10 nucleotides length were used with the forward primer. The number of cycles of denaturation, annealing and elongation was reduced to 15. The PCR products were visualised on a 1% (w/v) agarose gel and the bands of PCR products were excised from the gel and purified as described above. The amplified fragments with adapter and tags were quantified using a Qubit™ fluorometer (Invitrogen) and mixed in approximately equal concentrations (4 × 10^5^ copies μL^−1^). Pyrosequencing was performed on a GS FLX Standard PicoTiterPlate (70 × 75) using a GS FLX pyrosequencing system according to the manufacturer's instructions (Roche, Mannheim, Germany) at the Technical University of Copenhagen.

### Phylogenetic analysis of the isolates

16S rRNA gene sequences obtained from the isolates were assembled into contigs with ≥ 97% sequence similarity with the VectorNTI (Invitrogen) software and aligned using the SINA alignment service of the ARB-Silva database^35^. Each isolate sequence was complemented with closely related 16S rRNA gene sequences as determined by a BLAST^36^ search against the NCBI nucleotide database (19 July 2012). The nearest neighbours of the isolate sequences to be included in the tree were determined according to the following criteria: 1) all near neighbours that were previously found in association with marine invertebrates including the first free-living relative 2) if all the nearest neighbours were uncultured microorganisms, neighbours were added until a cultured relative was found, 3) in case the closest relative was a cultured, free-living microorganism, 3–5 nearest neighbours were added to the tree. Ambiguous regions of the alignment were systematically removed using the program Gblocks v.0.91b^37^. Default parameters were used, except allowing a minimum block length of 5 and gaps in 50% of positions. Phylogenetic trees were calculated by Bayesian analysis, using MrBayes v3.0b4 software (www.bioportal.uio.no)^38^. Phylogenetic trees were visualized and curated using ARB^39^.

### Phylogenetic analysis of pyrosequencing data

Pyrosequencing data were analysed using the QIIME 1.3.0 pipeline^40^. No denoising was applied, but low quality sequences were removed using default parameters. Briefly, i. reads with fewer than 200 or more than 1000 nucleotides were removed; ii. reads with more than 6 ambiguous nucleotides, homopolymer runs exceeding 6 bases, reads with missing quality scores and reads with a mean quality score lower than 25 were removed; iii. reads with mismatches in the primer sequence were removed, and operational taxonomic units (OTUs) were identified at the 97% identity level. Representative sequences from OTUs were aligned using PyNAST^41^. The taxonomic affiliation of each OTU was determined using the RDP Classifier at a confidence threshold of 80%^42^. Possible chimeric OTUs were identified using QIIME's ChimeraSlayer and removed from the initially generated OTU list, producing a final set of non-chimeric OTUs. All OTUs representing at least 1% of the reads in Crambe 1 or Crambe 3, and OTUs that were found to be closely related to the *C. crambe* isolates (BLAST analysis of the 16S rRNA gene sequences of isolates against a database containing the pyrosequencing OTUs at a cut-off of 97% using a locally installed version of Blast2-2-4^36^) were considered for a more thorough phylogenetic analysis. Representative sequences of the OTUs were aligned using the SILVA online SINA alignment service^35^ and were subsequently added to the Bayesian tree generated for the *C. crambe* isolates and their neighbours by use of the ARB parsimony method without changing the tree topologies.

### RNA extraction, RT-PCR, cloning and analysis of bacterial halogenase genes

Total RNA was extracted from Crambe 13 using the guanidine-thinocyanate-phenol-chloroform method^43^. 100 mg pieces of sponge tissue were removed from RNAlater and submerged in 1 ml of Solution D supplemented with 600 U/ml of Proteinase K. The tissue was disrupted by pipetting and extracted with phenol and chloroform. The RNA was dissolved in RNA Storage Solution (Ambion) after being treated with Turbo DNase Free (Ambion) and purified on RNeasy Minelute spin columns (Qiagen). RNA quantity and quality was determined spectrometrically using a NanoDrop™ 1000 Spectrophotometer (Thermo Fisher Scientific, Landsmeer, the Netherlands), and RNA integrity was determined with the Experion™ RNA StdSens Analysis Kit (BioRad, Veenendaal, the Netherlands).

RT-PCR targeting halogenase genes was performed with the primers H002 (forward) and H005 (reverse)^24^. The RT reaction (20 μl) contained 50 mM Tris-HCl, 75 mM KCl, 3 mM MgCl_2_, 5 μM DTT, 0.5 mM of each dNTP, 2 pmol of primer H005, 200 U of of SuperScript™ Reverse Transcriptase (Invitrogen), 40 U of RNasin® Plus RNase Inhibitor (Promega) and 1 μg of RNA. Reactions were incubated at 55°C for 60 minutes, followed by 15 minutes at 70°C to denature the reverse-transcriptase.

The PCR components were identical to those used to amplify the 16S rRNA genes, except that 1–3 μl of the RT reaction were used as template. When the sponge-derived isolates were screened for halogenases, 100 ng of gDNA were used as template. The PCR conditions were: initial denaturation (5 min, 95°C) followed by 30 cycles of denaturation (30 s, 94°C), primer annealing (45 s, 60°C), primer extension (50 s, 72°C), and a final extension (5 min, 72°C). For each reaction, the band of the right size (approx. 500 bp) was excised from the gel, purified and cloned into a PGEM-T Easy I vector (Promega) following manufacturer's protocol. Clones were screened for the presence and size of the insert using T7-Sp6 primers and those with the right size were sequenced as described above.

### Phylogenetic analysis of halogenase sequences

Sequence analysis of the clones was performed with an NCBI-BLASTx search against the NCBI Microbes database. Sequences which had a similarity to microbial halogenase encoding genes were translated into amino acid sequences using the ExPASy Translate tool^44^. These sequences as well as the nearest relatives determined by BLASTx and the bacterial halogenase gene sequences used by Bayer et al.^12^ were aligned using the T-Coffee multiple sequence alignment tool (www.tcoffee.org)^45^. The halogenase clone sequences from the aforementioned study were not included in the alignment as the PCR-amplified fragments had no overlap with the cDNA sequences in this study. A phylogenetic tree was calculated with the RaxML maximum likelihood tree calculator (http://www.trex.uqam.ca/)^46^. The Whelan and Goldman amino acid replacement matrix, Gamma substitution model and the hill-climbing algorithm were used. For each distinct starting tree, 100 bootstrap replicates were calculated. The final tree was visualized with FigTree V.2.

## Author Contributions

B.Ö., H.S. and D.S. designed the experiment. B.Ö. and L.d.J. conducted the experiments. B.Ö. and D.S. analysed the data and B.Ö., H.S. and D.S. wrote the paper. All authors discussed the results and commented on the manuscript at all stages.

## Figures and Tables

**Table 1:** Relative abundance of isolates in the sponge tissue as identified by pyrosequencing the partial 16S rRNA gene in Crambe1/Crambe3, respectively. The frequency indicates the number of times the isolates were obtained

isolate	frequency	OTU	% reads	phylum
isolate 2	17	2245	8.0/0.02	*Gammaproteobacteria*
13Mu4	1	2245	see isolate 2	*Gammaproteobacteria*
22E1	1	2385	0.06/0.4	*Alphaproteobacteria*
isolate 11	33	2400	0.01/0.01	*Alphaproteobacteria*
isolate 4	5	979	0/0.01	*Actinobacteria*
isolate 7	6	-	-	*Firmicutes*
isolate 8	3	-	-	*Firmicutes*
isolate 10	7	-	-	*Firmicutes*
isolate 3	9	-	-	*Gammaproteobacteria*
isolate 12	3	-	-	*Gammaproteobacteria*
22B11	1	-	-	*Gammaproteobacteria*
23D8	1	-	-	*Gammaproteobacteria*
isolate 5	3	-	-	*Alphaproteobacteria*
20A1	1	-	-	*Alphaproteobacteria*
isolate 9	5	-	-	*Alphaproteobacteria*
21A7orange	1	-	-	*Alphaproteobacteria*
24F5	1	-	-	*Alphaproteobacteria*
23E7	1	-	-	*Alphaproteobacteria*
23E10	1	-	-	*Alphaproteobacteria*
isolate 1	3	-	-	*Actinobacteria*
A7pink	1	-	-	*Actinobacteria*
13Mu5	1	-	-	*Actinobacteria*
22C5	1	-	-	*Actinobacteria*
23D12	1	-	-	*Actinobacteria*

## Notes

**Accession numbers**: 16S rRNA gene sequences of isolates were submitted to the NCBI GenBank Database under accession numbers JX575188 - JX575291 and JX998187. Halogenase cDNA sequences were submitted to the NCBI GenBank Database under accession numbers JX683128-JX683145. Pyrosequencing data were deposited at the European Bioinformatics Institute in the sequence read archive under sample accession numbers ERS178984 and ERS178985 (http://www.ebi.ac.uk/ena/data/view/ERP001839).
